# Supplementary material for: COZOID: contact zone identifier for visual analysis of protein-protein interactions
Source: BMC Bioinformatics. 2018 Apr 6;19:125. doi: 10.1186/s12859-018-2113-6 (PMC5889581; doi:10.1186/s12859-018-2113-6)
Supplement: Supplementary file 2 — Software build. Executable binary file of the software tool. (ZIP 118,784 kb) [file 12859_2018_2113_MOESM2_ESM.zip › cozoid/platform/docs/testng-6.5.1-javadoc.zip!/org/testng/ISuiteResult.html]

ISuiteResult (TestNG)


---


|  |  |  |
| --- | --- | --- |
| **Overview** \| **Package** \| **Class** \| **Tree** \| **Deprecated** \| **Index** \| **Help** | |  |
| **PREV CLASS**   **NEXT CLASS** | **FRAMES**    **NO FRAMES**     **All Classes** |  |
| SUMMARY: NESTED \| FIELD \| CONSTR \| METHOD | DETAIL: FIELD \| CONSTR \| METHOD |  |


---


## org.testng Interface ISuiteResult

**All Superinterfaces:**: java.io.Serializable

---

``` public interface ISuiteResult extends java.io.Serializable ```

This class represents the result of a suite run.

**Author:**
:   Cedric Beust, Aug 6, 2004

---

| **Method Summary** | |
| --- | --- |
| `java.lang.String` | `getPropertyFileName()` |
| `ITestContext` | `getTestContext()` |

**Method Detail**

### getPropertyFileName

```
java.lang.String getPropertyFileName()
```

:   **Returns:**: The name of the property file for these tests.

---


### getTestContext

```
ITestContext getTestContext()
```

:   **Returns:**: The testing context for these tests.


---


|  |  |  |
| --- | --- | --- |
| **Overview** \| **Package** \| **Class** \| **Tree** \| **Deprecated** \| **Index** \| **Help** | |  |
| **PREV CLASS**   **NEXT CLASS** | **FRAMES**    **NO FRAMES**     **All Classes** |  |
| SUMMARY: NESTED \| FIELD \| CONSTR \| METHOD | DETAIL: FIELD \| CONSTR \| METHOD |  |


---
